# Ratiometric electrochemical aptasensor based on ferrocene and carbon nanofibers for highly specific detection of tetracycline residues

**DOI:** 10.1038/s41598-017-15333-5

**Published:** 2017-11-07

**Authors:** Qingcui Xu, Zengning Liu, Jiayun Fu, Wenping Zhao, Yemin Guo, Xia Sun, Haiyun Zhang

**Affiliations:** 1 School of Agriculture and Food Engineering, Shandong University of Technology, No.12 Zhangzhou Road, Zibo, 255049 Shandong Province P.R. China; 2 Shandong Provincial Engineering Research Center of Vegetable Safety and Quality Traceability, No. 12 Zhangzhou Road, Zibo, 255049 Shandong Province P.R. China; 3 School of Mechanical Engineering, Shandong University of Technology, No.12 Zhangzhou Road, Zibo, 255049 Shandong Province P.R. China

## Abstract

A sensitive and efficient ratiometric electrochemical aptasensor was designed for tetracycline (TET) detection in milk. The ratiometric electrochemical aptasensor was constructed by integrating two aptasensors termed as aptasensor 1 and aptasensor 2. The aptasensor 1 was fabricated that based on ferrocene (Fc) and gold nanoparticles (AuNPs) nanocomposite. Meanwhile, the aptasensor 2 was prepared that based on carbon nanofibers (CNFs) and AuNPs nanocomposite. TET-aptamer was immobilized effectively onto screen-printed carbon electrodes (SPCEs) surface through forming Au-S bond between AuNPs and thiol of aptamer at 5′ end to construct the aptasensor 1 and aptasensor 2. And their detection results were calculated by ratio. Thus, the proposed ratiometric aptasensor solved the problem of low accuracy and large differences between batches. Under the optimized conditions, the TET was detected by differential pulse voltammetry (DPV). Taken advantage of ratio calculation, the as-prepared ratiometric aptasensor could detect TET quantitatively in the range of 10^−8^–10^−3^gL^−1^, with a detection limit of 3.3 × 10^−7^gL^−1^. Moreover, its applicability to TET-contaminated real samples (milk) showed an excellent agreement with the values determined by ultrahigh-performance liquid chromatography-tandem mass spectrometry (UPLC-ESI-MS/MS). With high sensitivity, accuracy and reliability, the developed ratiometric aptasensor held a great potential in TET detection for food safety.

## Introduction

Tetracycline (TET) antibiotic, a kind of broad spectrum antibiotics, is used not only for prevention and treatment of animal diseases in livestock and poultry industry, but also for promoting growth^1^. However, TET residues in foods of animal origin become serious problems nowadays, the residues pose many health threats to the consumers, e.g. allergic reactions, antimicrobial resistance and gastrointestinal disturbance and TET pigmentation teeth^2^. For instance, the use of antibiotics, especially used without following label directions, can result in drug residues in animal-origin foods such as milk.

In an effort to protect consumers, a number of analytical methods have been developed to monitor antibiotic residues in milk. Up to now, some methods such as high performance liquid chromatography (HPLC)^3–5^, LC coupled with tandem mass spectrometry (LC-MS/MS) and immunoassays^6^ have been commonly performed for antibiotic residues detection. Despite their accuracy and reliability, these methods have certain practical disadvantages such as complexity, high cost, and time consumption. The sensitive determination method with high accuracy is still a challenge in the practical applications. Therefore, a simple, rapid and accurate detection method of these antibiotic residues is highly desired for the food safety. Biosensors detection methods can substitute the current analytical methods by simplifying or eliminating sample preparation, thus decreasing the analysis time and cost.

Currently, TET detection strategies are trended toward development techniques based on aptamer sensing methods. Compared with antibody or other biological recognition elements, aptamers exhibited many unique advantages such as low cost, easy production, high thermal & chemical stability and reproducible synthesis^7–10^. Aptasensors have been broadly used in detection of cancer cells, organic molecules, and a variety of proteins^11^. Recently, we have constructed many aptasensors for TET detection based on the specific reaction between aptamers and targets. Although the aptasensor showed high sensitivity, it suffered from the problem of low accuracy and large differences between batches^12,13^. In order to overcome the problem, a ratiometric electrochemical aptasensor for highly specific detection of TET residues is introduced in this work.

In addition, a signal amplification is a key factor for the fabrication of aptasensor. More recently, enormous efforts have been devoted to the development of nanomaterials for the signal amplification. Among the various electron transfer mediators, ferrocene (Fc) has been widely used to develop biosensors. Its unique sandwich structure, aromaticity of unique oxidation-reducing, and good biological compatibility made it have a broad application prospects in electrochemical sensors, such as detection of pesticide residues^14^ and metal ions^15^. As one kind of novel carbon material, carbon nanofibers (CNFs) have been receiving much attention in practical applications, such as detection of carbendazim^16^ and ethyl paraben (EPB) in pharmaceutical and cosmetic products^17^. Notably, CNFs with the favorable features of large aspect ratio and three-dimensional porous networks can accelerate the electron transport, improving the electron transfer rate^18,19^. Moreover, the ultra-long one-dimensional nanostructure of CNFs also contributes to gold nanoparticles (AuNPs) deposition which can effectively reduce the charge diffusion length as well as improve the accessible surface area of AuNPs, being conducive to improve the electric conductivity. To the best of our knowledge, utilization of AuNPs-Fc and CNFs-AuNPs for the detection of TET residues via an aptasensing system has not been reported before.

As mentioned above, AuNPs-Fc and CNFs-AuNPs nanocomposite can form a unique sensing film with strong synergistic effects. Aptamers exhibit many unique advantages such as low cost, high thermal & chemical stability. Thus, we introduced a novel and highly accurate ratiometric aptasensor based on Fc and CNFs for sensitive detection of TET through an assembly strategy of the thiol functionalized aptamers and AuNPs. To the best of our knowledge, this kind of aptasensor has not yet been reported. Herein, a ratiometric electrochemical aptasensor was constructed by measuring the ratio of currents changes before and after the target combination (referred as ∆*I*_CNFs_/∆*I*_Fc,_ here, ∆*I*_CNFs_ refers to the current changes of the electrode modified with CNFs and AuNPs when detecting TET, ∆*I*_Fc_ refers to the current changes of the electrode modified with AuNPs and Fc when detecting TET). Finally, TET in real milk samples was analyzed with this ratiometric aptasensor. The method demonstrated a high sensitivity and accuracy. Thus, it may have potential applications for the detection of residual TET antibiotics in the field of food analysis.

## Experimental

### Apparatus

Cyclic voltammetry (CV) and DPV measurements were performed with a CHI660D electrochemical workstation (Shanghai Chenhua Co., China). All experiments were performed with a three-electrode system at room temperature. The commercially available screen-printed carbon electrodes (SPCEs (TE100, working diameter was 3 mm) were purchased from Zensor R&D (Taiwan). The morphologies of modified electrodes were observed by a scanning electron microscope (SEM, Netherlands). TET in real milk samples was also detected by UPLC-ESI-MS/MS. The solution pH values were measured using an FE 20 K Mettler-Toledo pH meter (Switzerland). Ultrasonication was performed using a SK3300H ultra-sonic cleaner (Shanghai, China). The solution was blended using a PTR-35 SPC vortex mixer (Britain).

### Reagents and materials

NaH_2_PO_4_·2H_2_O and Na_2_HPO_4_·12H_2_O were purchased from Beijing Chemical Technology Co., Ltd. (Beijing, China). The 0.1 M pH 7.5 phosphate buffer solutions (PBS) were prepared by mixing the stock solutions of NaH_2_PO_4_·2H_2_O and Na_2_HPO_4_·12H_2_O. Fc was purchased from Hongyan Chemical Reagent Factory (Tianjin, China). CNFs were obtained from Beijing Gold Deco Island Co., Ltd. (Beijing, China). K_3_[Fe(CN)_6_] and K_4_[Fe(CN)_6_] were purchased from Yongda Chemical Reagent Co., Ltd. (Tianjin, China). The specific aptamer (Apt) sequences for TET were identified by Aniela Wochner *et al*. DNA oligonucleotides modified with mercapto groups (5′-SH-(CH_2_)_6_-CGT ACG GAA TTC GCT AGC CCC CCG GCA GGC CAC GGC TTG GGT TGG TCC CAC TGC GCG TGG ATC CGA GCT CCA CGT G-3′) were synthesized by Sangon Biotech Co., Ltd. (Shanghai, China). TET was obtained from the Sigma Company (USA). All other chemicals were of analytical reagent grade. All the solutions were prepared with ultrapure water (18.2 MΩ·cm) which was purified with a LS MK2 PALL purification system (PALL, USA).

### Preparation of AuNPs/AuNPs-chitosan (CS) composites

Prior to fabricating composites, all glassware used in the preparation was thoroughly cleaned in aqua regia (HCl:HNO_3_ = 3:1), rinsed in triply ultrapure water and oven-dried prior to use. 100 mL of 0.01% HAuCl_4_ (w/v) aqueous solution was added in a flask. The flask was placed on an electric furnace for heating, with violent stirring until the solution was boiled, and then 2.5 mL 1% lemon acid sodium solution was quickly added. Accompanying with the reduction reaction, the solution quickly was turned into a ruby red color, indicating the formation of the AuNPs. After stirred continuously and vigorously for 15 min, the solution was cooled to room temperature. The resulting solution was then stored in a brown bottle and kept at 4 °C. The resulting nanocomposites were used for all the characterizations and experiments.

0.1% (w/v) chitosan (CS) solution was prepared by dissolving 0.1 g CS into 100 mL 1.0% acetic acid and stirring for 4 h. Then the CS solution was added into the as-prepared AuNPs solution, and after being stirred for 1 h AuNPs-CS solution was prepared. The resulting solution was stored in refrigerator and kept at 4 °C.

### Preparation of Apt/Fc-Apt composites

According to the illustrations of the TET-aptamer, 14 mL buffer (0.1 M PBS, pH 7.0) was added into an OD primer and formatted into 100 μM storage liquid. The aptamers were easily attached to the wall of the tubes, so it was needed to be centrifuged (10 000 rpm) for 5 minutes before opening the tube. In this work, 140 mL of 0.1 M PBS (pH 7.0) was respectively added into each OD tube and configured to a 10 μM TET-aptamers. Various concentrations of TET-aptamers were obtained by the dilution of the 10 μM TET-aptamer for the follow-up experiments. The resulting aptamer was preserved at −20 °C when not in use.

1% (w/v) Fc solution was prepared by dissolving 1 g Fc into 100 mL ethanol solution and sonicating for 30 min. Then the Fc solution was added into the as-prepared different concentrations of TET-aptamer by stirring for 12 h at 4 °C, and then the aptamers labeled with Fc was obtained by the groove-face interaction.

### Preparation of the aptasensor

Prior to modification, a potential of +1.75 V was applied to the SPCE, with stirring, in pH 5.0 PBS for 300 s and the electrode was then scanned from +0.3 V to +1.25 V and from −1.3 V to +0.3 V until a steady state current-voltage curve was obtained. The pretreated SPCEs were used for the following experiments^20^.

Fabrication of the AuNPs-CS/Fc-Apt/BSA/TET aptasensor 1 is as follows: Firstly, 7 μL of the AuNPs-CS nanocomposites was pipetted onto the surface of the SPCEs, the modified electrode was dried at room temperature. Then, 7 μL of the aptamer labeled Fc was assembled on the above-modified electrode surface. Following that, the electrode was incubated with 0.5% bovine serum albumin (BSA) for 30 min in order to block possible remaining active sites and avoid non-specific adsorption. Finally, the target TET was added dropwise onto the modified electrode surface and incubated for 1 h.

Fabrication of the CNFs/AuNPs/Apt/BSA/TET aptasensor 2 is as follows: Firstly, 7 μL of the CNFs solution was pipetted onto the surface of the SPCEs. Then, 7 μL of the AuNPs was added. Following that, aptamers modified with thiol were assembled on the above-modified electrode surface. Then, the electrode was incubated with 0.5% BSA for 30 min. Finally, the target TET was added dropwise onto the modified electrode surface and incubated for 1 h.

**Figure 1:**
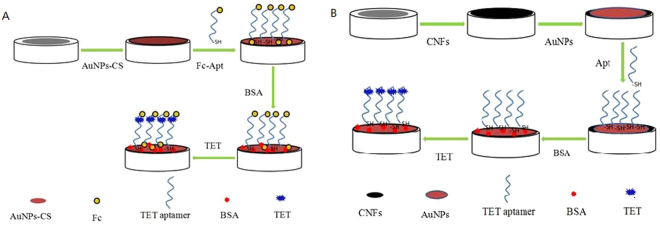
Assembly processes of the ratiometric electrochemical aptasensor.

The finished aptasensor was stored at 4 °C when not in use. The stepwise fabrication procedure of aptasensor was shown in Fig. 1A,B.

### Electrochemical measurements

The composites of AuNPs-CS, CNFs and Fc-Apt were characterized by an scanning electron microscopy (SEM). All electrochemical measurements were performed in 15 mL 0.1 M PBS (pH 7.0) containing 5 mM K_3_[Fe(CN)_6_]/K_4_[Fe(CN)_6_] (1:1 mixture as a redox probe) and 0.1 M KCl at room temperature. CV was performed over a potential range from −0.2 to 0.6 V at a scan rate of 50 mV/s. EIS (electrochemical impedance spectroscopy) was were carried out in PBS (pH7.0) containing 5 mM K_3_[Fe(CN)_6_]/K_4_[Fe(CN)_6_] (1:1) and 0.1 M KCl. The frequency was measured from 1 Hz to 100 KHz and the AC voltage amplitude was 55 mV. The electrochemical DPV measurements were carried out under the following conditions: The voltage was scanned from −0.05 V to 0.4 V with a pulse height of 50 mV, the step height and the frequency were kept as 4 mV and 15 Hz, respectively. The sensitivity and the specificity of the proposed electrochemical aptasensor were investigated by DPV. In addition, parameters affecting the aptasensor response such as pH of the base solution, the concentration of the aptamer and incubation time were optimized. After the optimization, the proposed aptasensor was applied for the detection of TET.

## Results and Discussion

### SEM characterizations of modified electrodes

**Figure 2:**
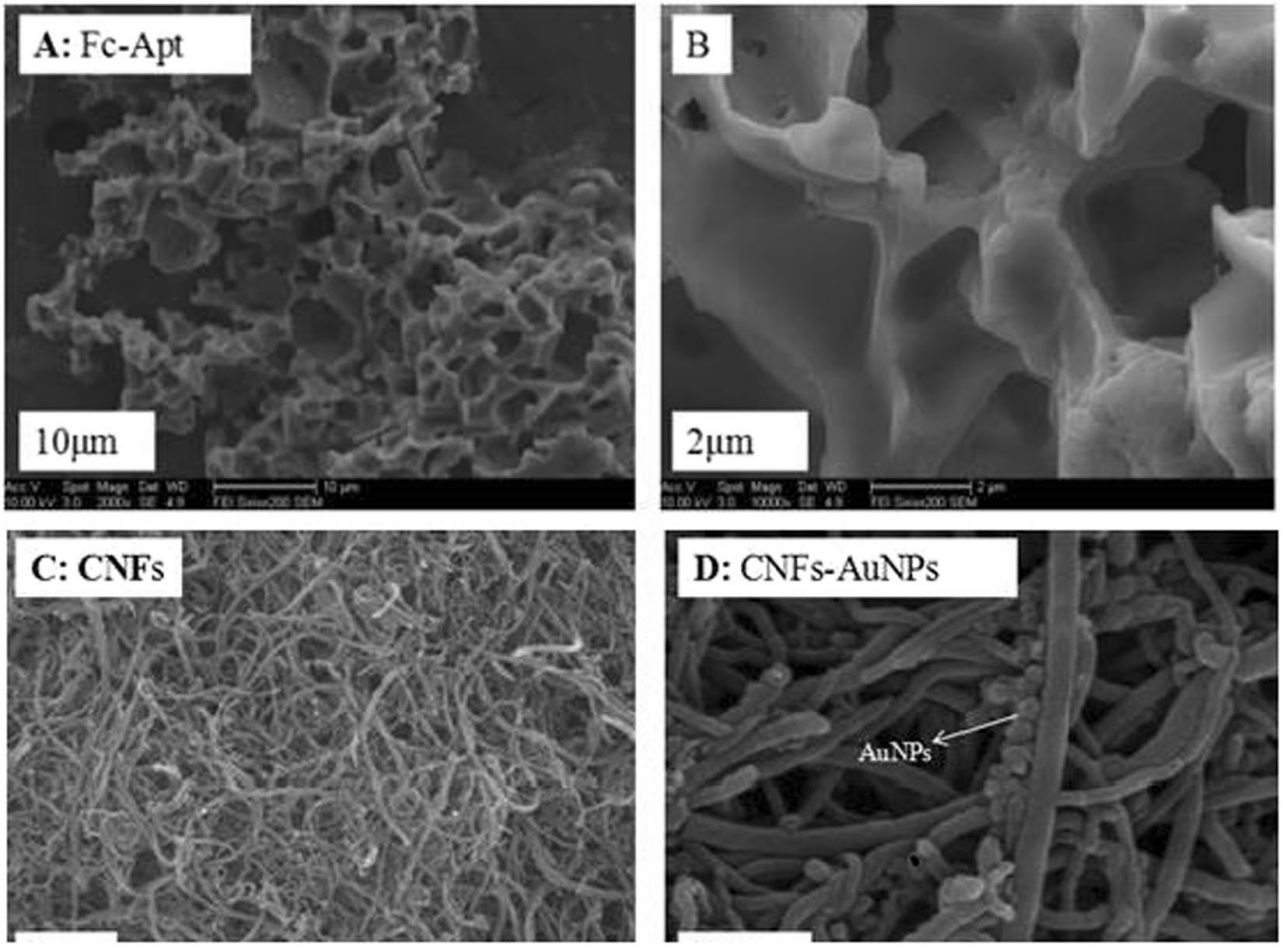
SEM images of Fc-Apt (**A**) 10 μm; (**B**) 2 μm. SEM images of CNFs (**C**) 1μm; CNFs-AuNPs (**D**) 200 nm.

**Figure 3:**
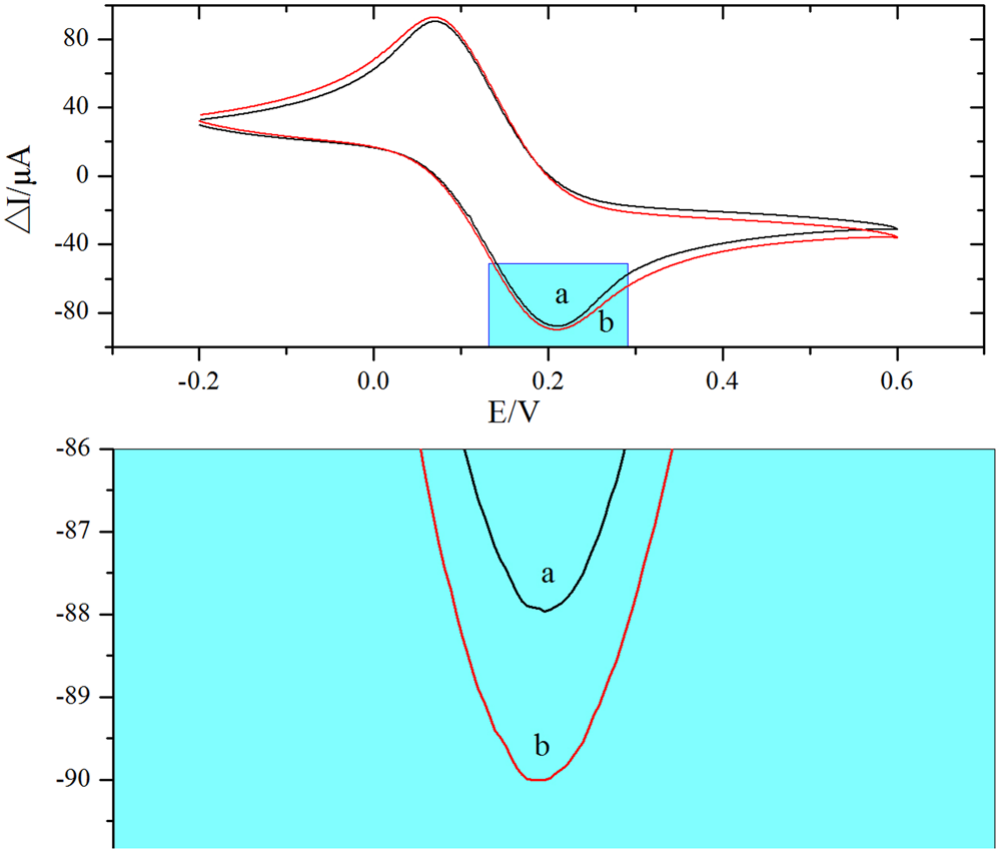
CVs of SPCEs modified with Fc-Apt (a)/Fc (b) and its partial enlarged detail. (recorded in 0.1 M PBS (pH 7.0) containing 5.0 mM [Fe(CN)_6_]^3−/4−^ and 0.1 M KCl, the scan rate was 50mVs^−1^).

The morphologies and microstructures of the as-prepared different films were studied by SEM observation (Fig. 2). As can be seen from the Fig. 2A, the aptamers and Fc were well mixed together. Figure 2B showed that the Fc-Apt in more and more detail, having zoomed in further with the scanning electron microscope. We can see from the Fig. 2B, the aptamers and Fc formed a very good groove combination. Combined with the CD spectrum data reported in the literature^21^, the distance of two cyclopentadiene ring of Fc was 0.336 nm and Fc was difficult to combine with the aptamer through the insert mode^22–24^. Fc may be related to the aptamer molecule through the accumulation and formed a very good groove combination^25^. Moreover, when Fc was coated by the aptamer, the electrochemical activity of the electrode surface was reduced and there is decreasing in the oxidation peak current. And, as can be seen from the partial magnification of Fig. 3, the reduction peak current of Fc-Apt was increased (Fig. 3).

Figure 2C was the characterization of CNFs, which have many edge sites on the outer wall without any hollow cores. After adding AuNPs, we can see from the Fig. 2D, AuNPs were successfully attached to the surface of CNFs and the ultra-long one-dimensional nanostructure of CNFs also contributes to nanostructured AuNPs depositing, which can effectively reduce the charge diffusion length as well as improve the accessible surface area of AuNPs, being conducive to improve the electrical conductivity.

### Electrochemical behavior of the modified electrodes

**Figure 4:**
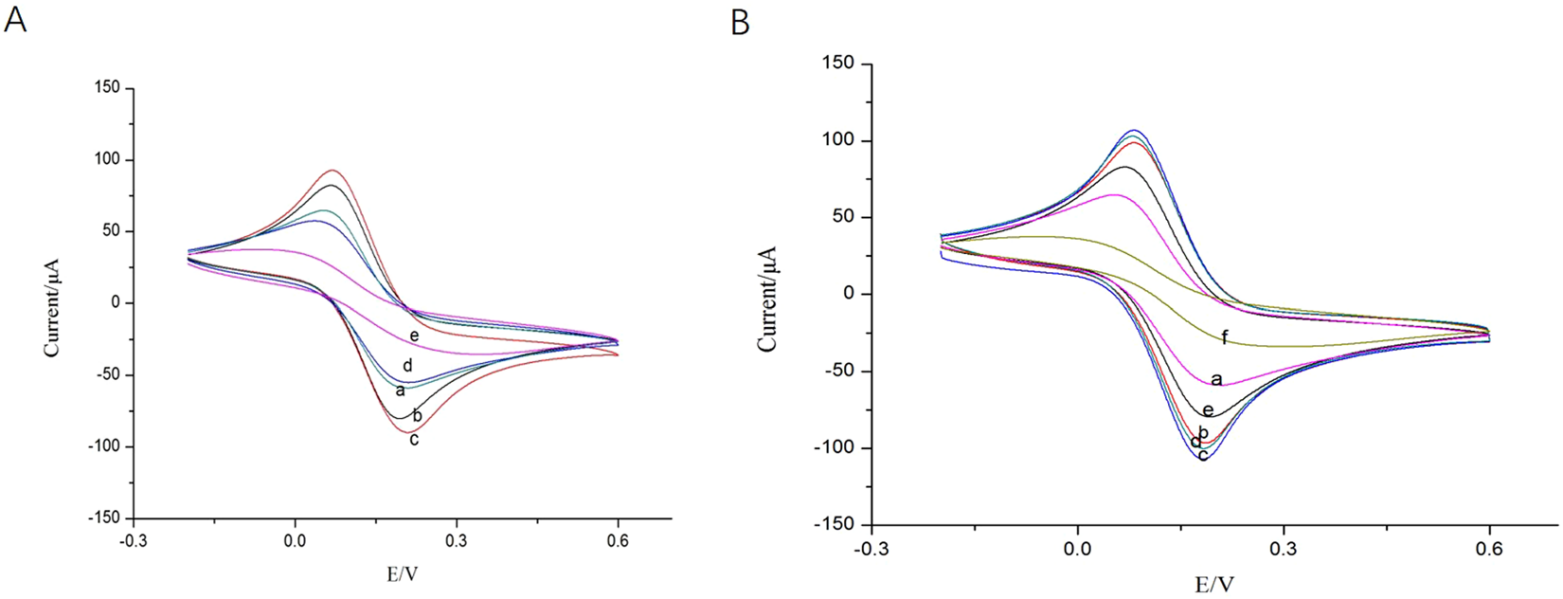
CVs of the stepwise assembly of the aptasensor recorded in 0.1 M PBS (pH 7.0) containing 5.0 mM [Fe(CN)_6_]^3−/4−^ and 0.1 M KCl, the scan rate was 50 mVs^−1^: (**A**) a bare SPCE; b AuNPs-CS/SPCE; c Fc-Apt/AuNPs-CS/SPCE; d BSA/Fc-Apt/AuNPs-CS/SPCE; e TET/BSA/Fc-Apt/AuNPs-CS/SPCE; (**B**) a bare SPCE; b CNFs/SPCE; c AuNPs/CNFs/SPCE; d Apt/AuNPs/CNFs/SPCE; e BSA/Apt/AuNPs/CNFs/SPCE; f TET/BSA/Apt/AuNPs/CNFs/SPCE.

The stepwise assembly of the aptasensor was characterized by CV and EIS. The results were shown in Fig. 4A and B. As can be seen from Fig. 4A, the bare SPCEs has an obvious redox peak (curve a). After the AuNPs-CS was immobilized on the bare electrode, a larger current response was exhibited (curve b) and the current increased to 80.48 μA. When aptamer (6 μM) labeled Fc was modified on the above-modified electrode surface, the peak current continued to increase to 90.75 μA (curve c), which suggested that Fc enhanced a current signal. After BSA was modified on the electrode, the peak current decreased to 55.39 μA (curve d) and probably because BSA blocked non-specific sites, reduced the effective area and functional sites for electron transfer. When 100 μg/L TET was added on the electrode, the peak current further decreased to 36.21 μA (curve e), because that the reaction of the aptamer and TET produced a large molecular substance that is not electrically conductive. This result demonstrated that AuNPs exhibit excellent conductivity in improving electron transfer, which was probably due to the good biocompatibility of AuNPs to preserve effectively the activity of the aptamers during preparation of the electrode.

**Figure 5:**
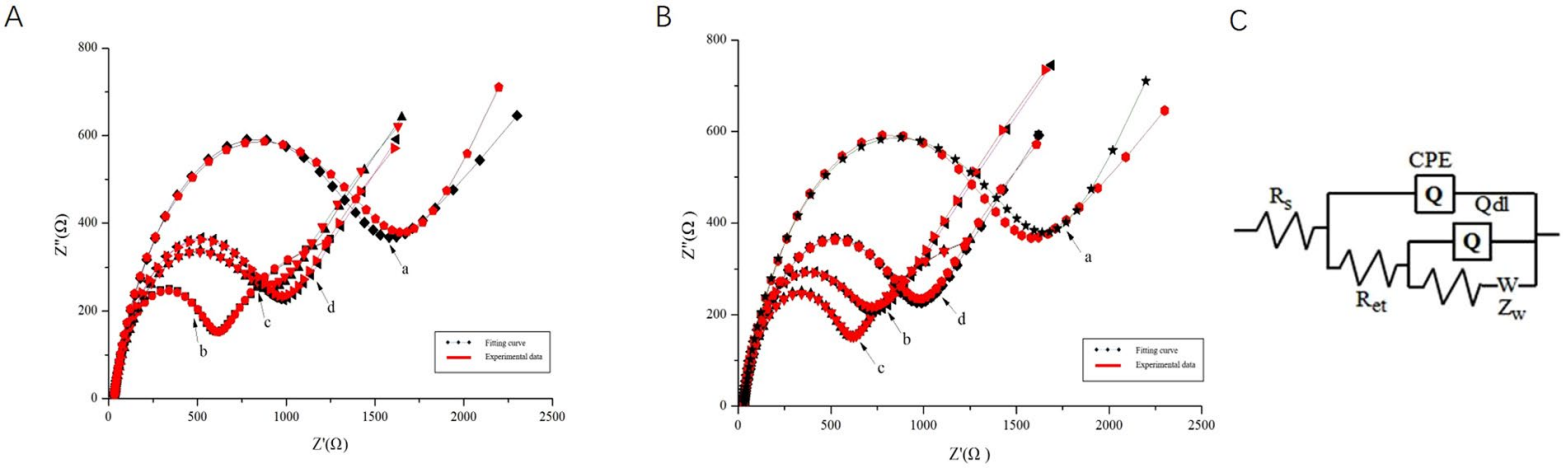
EIS of the stepwise assembly of the aptasensor: (the black line was fitting curves, the red line was experimental data) (**A**) a bare SPCE; b AuNPs-CS/SPCE; c BSA/Fc-Apt/AuNPs-CS/SPCE; d TET/BSA/Fc-Apt/AuNPs-CS/SPCE; (**B**) a bare SPCE; b CNFs/SPCE; c AuNPs/CNFs/SPCE; d BSA/Apt/AuNPs/CNFs/SPCE; e TET/BSA/Apt/AuNPs/CNFs/SPCE; (**C**) Equivalent circuit: R_s_: electrolyte resistance; Q_dl_: double layer capacitance; Z_W_, Warburg impedance; R_et_, electronic transfer interface oxidation reduction reaction.

Similarly, as can be seen from Fig. 4B, the peak current increased to 96.24 μA after CNFs was modified on the surface of SPCEs (curve b), which revealed that the CNFs film can be functioned as an electron-conducting tunnel. Furthermore, after coated with AuNPs, the peak current of obtained electrode increased again (curve c), which indicated that the AuNPs film could promote the electron transfer between the electrode surface and [Fe(CN)_6_]^3−/4−^. However, the redox peaks decreased obviously when aptamer (curve d) and BSA (curve e) were modified onto the electrode, attributing to their non-electrochemical activity, which partially blocked the electron transfer between the [Fe(CN)_6_]^3−/4−^ solution and the electrode. When 100 μg/L TET was added on the electrode, the peak current further reduced to 34.15 μA (curve f). Results of EIS agreed with those of the CVs (Fig. 5).

### Optimization parameters of the aptasensor performance

**Figure 6:**
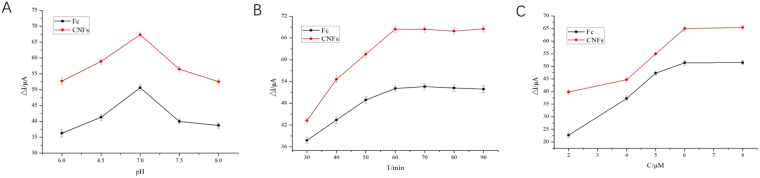
(**A**) Optimization of pH; (**B**) Optimization of aptamer concentration; (**C**) Optimization of incubation time.

To further optimize the developed aptasensor for quantification of the TET, optimization of the pH, aptamer concentration and incubation time were determined to optimize the aptasensor signal, and the corresponding results are presented in Fig. 6.

The pH value plays an important role in achieving excellent analytical performance. The response currents changes before and after the target combination of the modified electrodes were investigated in a series of PBS (0.1 M, pH from 6.0 to 8.0) including 5 mM K_3_[Fe(CN)_6_]/K_4_[Fe(CN)_6_] and 0.1 M KCl (as shown in Fig. 6A). The detection concentration of the TET was 1 mg/L. It can be seen from the graph that the aptasensor peak current value increases with the increase of the pH value, the maximum value of the peak current was at pH 7.0. The peak current showed a trend of decreasing when the pH value of the base solution continued to rise, which indicated that the pH of the basic solution has a great influence on the performance of the aptasensor. This is explained by the fact that the formation of complexes with TET and aptamer can be dissociated in alkaline environment or weak acidic environment. Thus, pH 7.0 of base solution was used in the subsequent experiment.

The influence of aptamer concentration on the response of the aptasensor was also studied (as showed in Fig. 6B). The detection concentration of the TET was 1 mg/L. The results showed that the peak current increased gradually as the aptamer concentration increased and reached a maximal value at 6 μM. After that, the response was almost stable as the concentration of the aptamer further increased, which shows that the accounted aptamer fixed on the aptasensor has reached a saturation point. Therefore, 6 μM of aptamer was chosen as the optimum aptamer concentration for fabrication of the aptasensor.

The effect of incubation time was investigated under the above optimal experimental parameters. The detection concentration of the tetracycline was 1 mg/L. The results showed that the peak current increased greatly with the increase of incubation time (as showed in Fig. 6C). When the time was longer than 60 min, the incubation curve moved to a stable value, which indicated that the interaction between TET and TET-aptamers had reached equilibrium. Thus, the time of 60 min was selected as the optimal incubation time.

### DPV response and calibration curve

**Figure 7:**
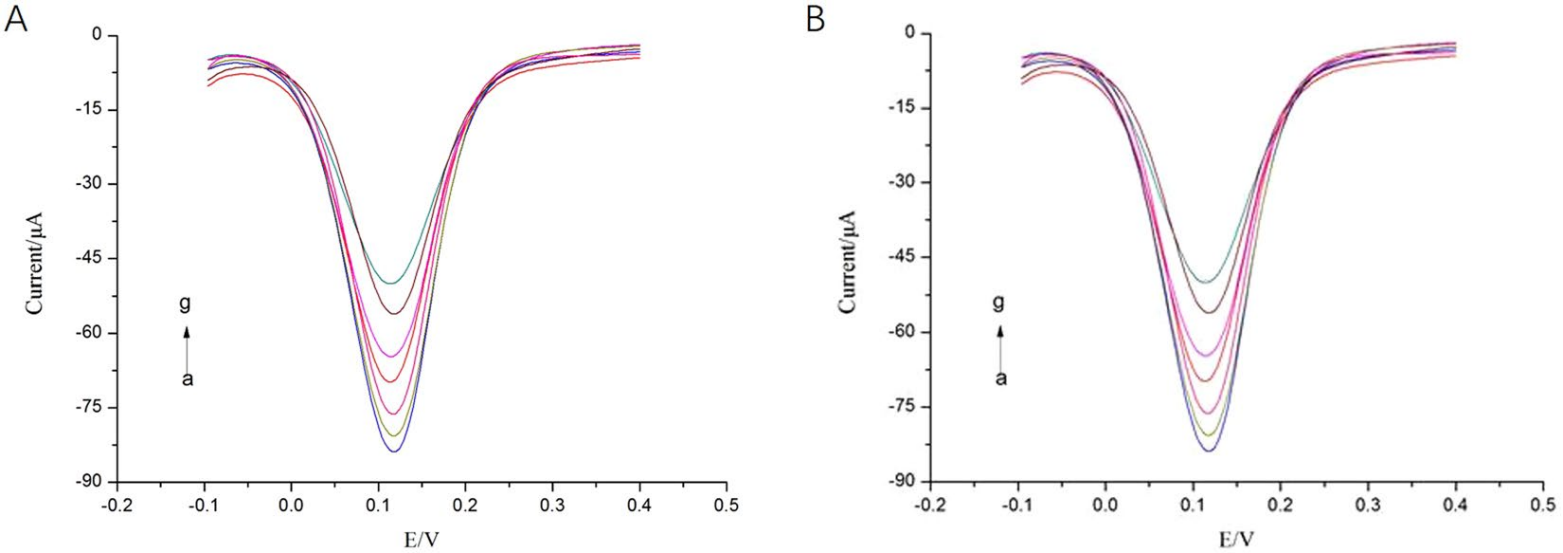
DPV responses of the two aptasensors at tetracycline with different concentrations (from a to g): currents change with 10^−8^ g/L to 10^−3^ g/L, A and B: a.0 g/L, b.10^−8^ g/L, c.10^−7^ g/L, d.10^−6^ g/L, e.10^−5^ g/L, f.10^−4^ g/L, g.10^−3^ g/L.

**Figure 8:**
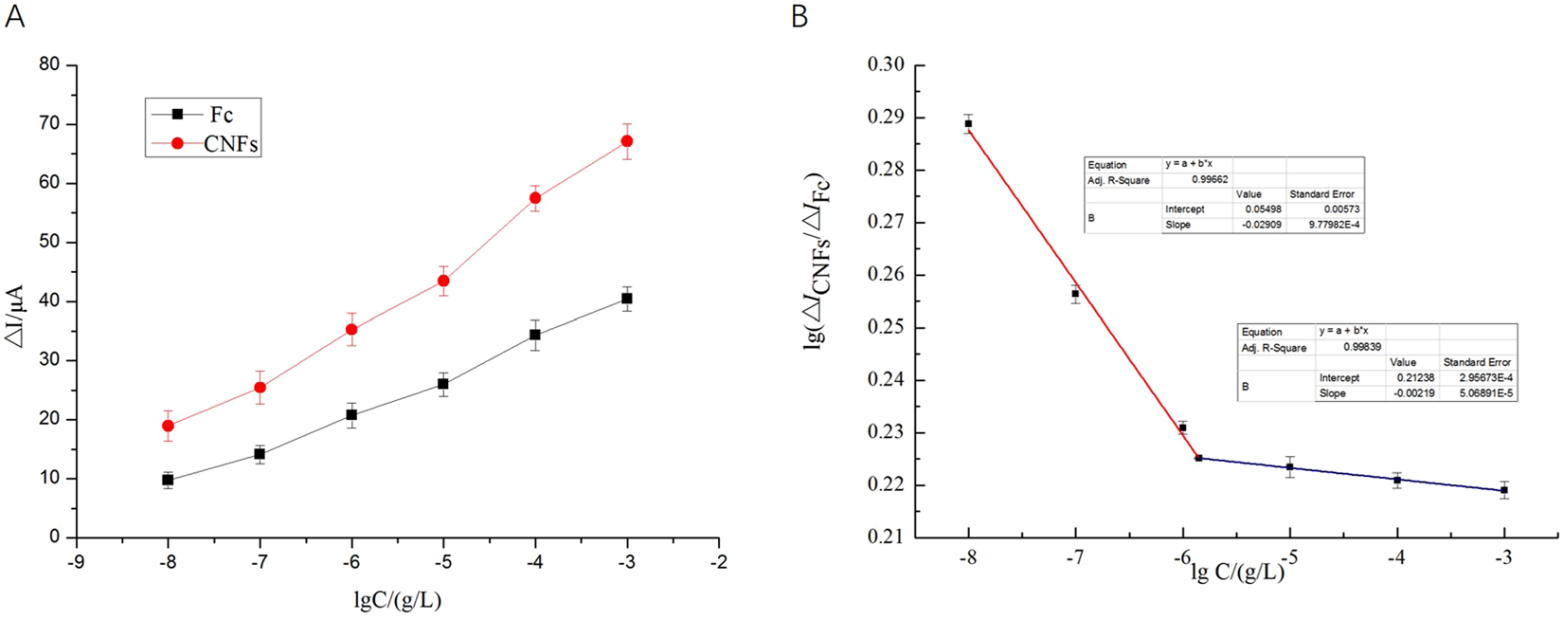
(**A**) Relationship between the currents changes and the logarithm concentration of tetracycline: the aptasensor1: aptamers labeled with ferrocene; the aptasensor2: modified with carbon nanofibers. (**B**) The calibration curve between the logarithm of the ratio (△*I*_CNFs_/△*I*_Fc_) and the logarithm of tetracycline concentration.

Different concentrations of TET was detected by the above two prepared aptasensors under the above optimal experimental parameters, and three parallel tests were made for each concentration of TET. Current changes from 10^−8^ to 10^−3^g/L of TET concentration were shown in Fig. 7A,B. As showed in Fig. 7A, current changes of the aptasensor 1 increased with the increase of TET concentration. Similarly, a gradual increase in current changes was observed with increasing TET concentration by using the aptasensor 2 modified with CNFs (as showed in Fig. 7B). The corresponding changes of the current response (Δ*I* = *I*_sample_ − *I*_control_) of the two aptasensors were shown in Fig. 8A in the linear range from 10^−8^ to 10^−3^ gL^−1^. Figure 8A presented the relationship between the currents changes and the logarithm of TET concentration. It could be found that the currents changes of the two aptasensors based on CNFs or Fc were positively related to logarithm of TET concentration.

In order to achieve high accuracy, sensitivity and decrease the difference between batches, our results ultimately set up the strategy of a ratiometric electrochemical aptasensor. We used logarithm of the ratio (∆*I*_CNFs_/∆*I*_Fc_) as ordinate and logarithm of TET concentration as abscissa to make the calibration curve (Fig. 8B). Figure 8B depicted the logarithm of the ratio (∆*I*_CNFs_/∆*I*_Fc_) showed a linear relationship with the logarithm of TET concentration in the range of 10^−8^–10^−6^ gL^−1^ and 10^−6^–10^−3^ gL^−1^. Thus, the use of ∆*I*_CNFs_/∆*I*_Fc_ ratio has better detection properties than ∆*I*_CNFs_ or ∆*I*_Fc_. The quantitative TET detection could be achieved according to the linear equation of y = 0.05498–0.02909x (R^2^ = 0.996, range of 10^−8^–10^−3^ gL^−1^) and y = 0.21238–0.00219x (R^2^ = 0.998, range of 10^−6^–10^−3^ gL^−1^) with a detection limit of 3.3 × 10^–7^ g/L (S/N = 3). Compared with other previously reported methods of detecting TET, the proposed ratiometric electrochemical aptasensor exhibited a higher sensitivity with a lower detection limit (see the Table 2).

### Specificity, repeatability and stability of the ratiometric aptasensor

**Figure 9:**
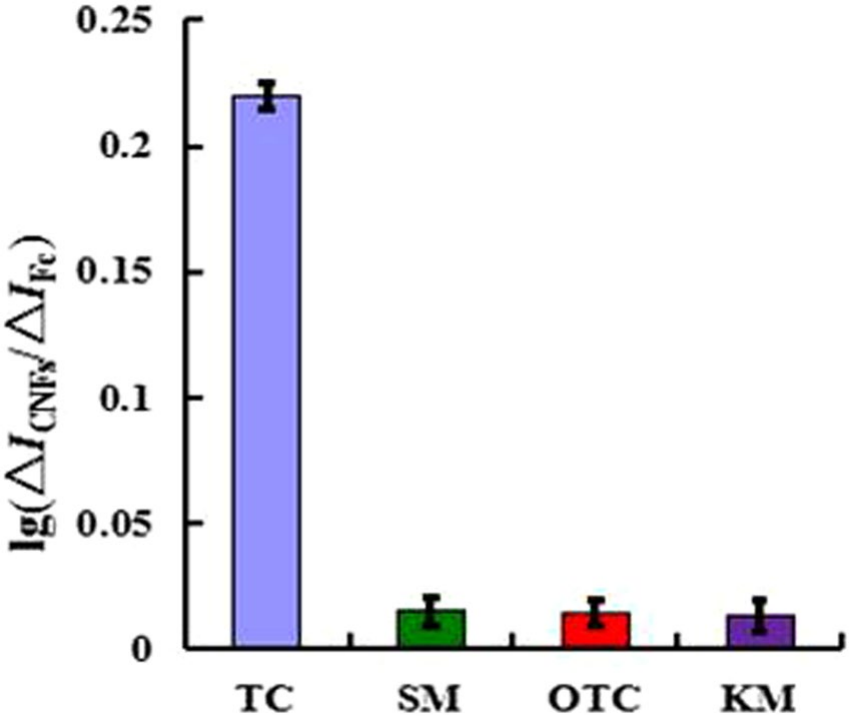
Specificity of the aptasensor detection of tetracycline (100 μgL^−1^) against other non-target small molecule antibiotics: SM (100 μgL^−1^), OTC (100 μgL^−1^), and KM (100 μgL^−1^).

A specificity is an important property of an aptasensor. The aptasensor was evaluated for specificity in pH 7.0 PBS containing 5 mM [Fe(CN)_6_]^3−/4−^ and 0.1 M KCl by testing other non-target small molecule antibiotics such as streptomycin (SM), oxytetracycline (OTC), and kanamycin (KM). The testing concentration was 100 μgL^−1^. The current responses of different antibiotics were obtained (Fig. 9). It was observed that the current change of the other three antibiotics was negligible, indicating that the specificity of the developed ratiometric electrochemical aptasensor for TET was good.

To investigate the repeatability of the aptasensor, five aptasensors fabricated independently under the same conditions were examined. The tested TET concentration was 100 μgL^−1^. Each of the five aptasensors was used 3 times continuously, and the average value of the obtained impedance difference in five groups was analyzed. The inter- and intra-group coeffcients of variation was 3.4% and 4.62%, which indicated that the ratiometric electrochemical aptasensor had good repeatability.

A stability is a key parameter for the application and development of the sensor. Under optimized optimal conditions, we prepared four aptasensors and stored them for 15 days at 4 °C. Then the above aptasensors were utilized to detect μgL^−1^ of TET. The current response decreased by about 6.2%, demonstrating that the ratiometric electrochemical aptasensor had good stability.

### Determination of tetracycline in real samples

**Figure 10:**
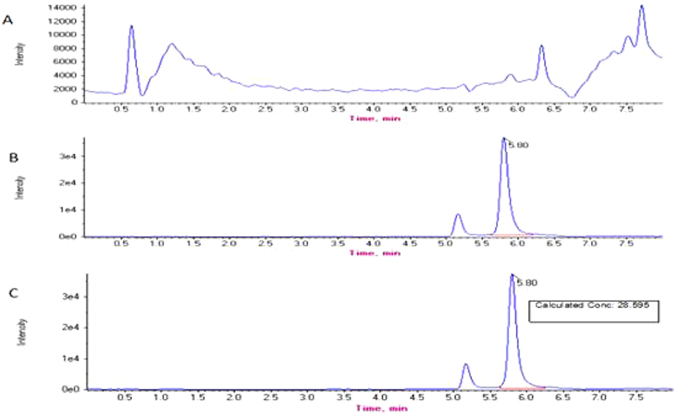
The detection results of tetracycline residues in milk samples using UPLC-ESI-MS/MS.

Although the proposed ratiometric electrochemical aptasensor showed good selectivity towards TET, it is worth exploring the analytical utility of the aptasensor for practical application. The milk samples used in this work were all purchased from a supermarket in China. The proportion of the original fat in the milk was 6%. Preprocessing: the milk sample was diluted according to a dilution ratio of 1:10, and then centrifuged at 20 000 rpm for 90 min. Finally, the milk was divided into three layers. The macromolecular material in the upper and lower layer, such as fat and casein, was removed. Furthermore, the TET standard solution was spiked into the diluted milk, making concentrations of 0, 5 × 10^−7^, 5 × 10^−6^, 5 × 10^−5^, 5 × 10^−4^ gL^−1^, and then experiments were carried out according to the aforementioned optimized conditions for TET detection with the developed aptasensor. As a result, the TET concentration recoveries were between 95.98% and 104.28% (Table 1), which clearly indicated that the aptasensor was suitable for the detection of TET in real milk samples. The detection results of UPLC-ESI-MS/MS were shown in Fig. 10. And Fig. 10A shows the background spectra of blank milk samples, Fig. 10B shows the spectra of standard TET samples and Fig. 10C shows the spectra of milk added TET (3 × 10^−5^ g/L).

**Table 1:** Comparison with other reported methods of detecting tetracycline.

Methods of detection	Detection limit (g/L)	Liner range (g/L)	Ref.
ELISA Multi-detection	6.5 × 10^−6^	3 × 10^−7^–3 × 10^−5^	Liu *et al*.
CGS	4.0 × 10^−5^	0–5 × 10^−4^	Wang *et al*.
ELAA	2.5 × 10^−9^	1 × 10^−9^–1 × 10^−5^	Zhang *et al*.
Synchronous Fluorescence	2.5 × 10^−8^	1 × 10^−6^–1 × 10^−5^	Mao *et al*.
UPLC-ESI-MS/MS	1.0 × 10^−9^	1 × 10^−9^–1 × 10^−6^	Yue *et al*.
Ratiometric Aptasensor	3.3 × 10^−10^	1 × 10^−11^–1 × 10^−3^	This work

From Table 2 and Fig. 10, it can be seen that the recoveries of the ratiometric electrochemical aptasensor in this work and UPLC-ESI-MS/MS were both greater than 90%. The results demonstrated that this method was highly accurate, precise and reproducible. It can be used for direct analysis of practical samples.

**Table 2:** Testing results of tetracycline in milk samples.

Milk Samples	Blank Detection(g/L)	Added (g/L)	Standard Value(lg(I_CNFs_/I_Fc_))	Detection Value(lg(I_CNFs_/I_Fc_))	Recovery (%)	RSD (%)
1	Not detected	5 × 10^−7^	0.23890	0.22930	95.98	5.6
2	Not detected	5 × 10^−6^	0.22406	0.23364	104.28	5.6
3	Not detected	5 × 10^−5^	0.22181	0.21701	97.84	4.3
4	Not detected	5 × 10^−4^	0.21956	0.21156	96.36	3.5

## Conclusions

In conclusion, a ratiometric electrochemical aptasensor for TET detection was developed based on Fc and CNFs in this study. With the two aptasensors based on Fc and CNFs, the current changes ratio was measured for a ratiometric electrochemical detection of TET. In this work, the AuNPs-Fc and CNFs-AuNPs were not only used as nanocarriers to immobilize aptamers, but also used as electroactive materials for signal amplification. Compared with common electrochemical detection methods, the ratiometric electrochemical aptasensor provides the advantage of a decrease of difference among batches and high sensitivity & accuracy. In addition, the proposed ratiometric aptasensor was successfully applied for TET detection in milk samples. It would be a promising tool for use in food analysis and clinical diagnosis. Thus, we anticipate that this principle will open up a new opportunity for other analytes detection.
